# Mathematical Model of Ultrasound Attenuation With Skull Thickness for Transcranial-Focused Ultrasound

**DOI:** 10.3389/fnins.2021.778616

**Published:** 2022-02-17

**Authors:** Jiande Guo, Xizi Song, Xinrui Chen, Minpeng Xu, Dong Ming

**Affiliations:** ^1^Academy of Medical Engineering and Translational Medicine, Tianjin University, Tianjin, China; ^2^Department of Biomedical Engineering, College of Precision Instruments and Optoelectronics Engineering, Tianjin University, Tianjin, China

**Keywords:** transcranial focused ultrasound, attenuation, mathematical model, neuromodulation, neuroimaging

## Abstract

Transcranial-focused ultrasound (tFUS) has potential for both neuromodulation and neuroimaging. Due to the influence of head tissue, especially the skull, its attenuation is a key issue affecting precise focusing. The objective of the present study was to construct a mathematical model of ultrasound attenuation inclusive of skull thickness. First, combined with real skull phantom experiments and simulation experiments, tFUS attenuation of different head tissues was investigated. Furthermore, based on the system identification method, a mathematical model of ultrasound attenuation was constructed taking skull thickness into account. Finally, the performance of the mathematical model was tested, and its potential applications were investigated. For different head tissues, including scalp, skull, and brain tissue, the skull was found to be the biggest influencing factor for ultrasound attenuation, the attenuation caused by it being 4.70 times and 7.06 times that of attenuation caused by the brain and scalp, respectively. Consistent with the results of both the simulation and phantom experiments, the attenuation of the mathematical model increased as the skull thickness increased. The average error of the mathematical model was 1.87% in the phantom experiment. In addition, the experimental results show that the devised mathematical model is suitable for different initial pressures and different skulls with correlation coefficients higher than 0.99. Both simulation and phantom experiments validated the effectiveness of the proposed mathematical model. It can be concluded from this experiment that the proposed mathematical model can accurately calculate the tFUS attenuation and can significantly contribute to further research and application of tFUS.

## Introduction

Ultrasound technique is a significant development in the field of traditional diagnostic medicine and has a wide range of clinical applications (Kranjec et al., 2014; Jana et al., 2020). In recent years, transcranial focused ultrasound (tFUS) has gained wide attention in the fields of neuromodulation (Tufail et al., 2010; Deffieux et al., 2013; Mueller et al., 2014; Lee et al., 2016; Legon et al., 2018) and neuroimaging (Song et al., 2021; Zhou et al., 2021). Compared with existing neuromodulation techniques, such as deep brain stimulation and transcranial magnetic stimulation, tFUS has some advantages (O’Shea and Walsh, 2007; Oluigbo et al., 2012; Mueller et al., 2017). First, tFUS is a non-invasive method that does not require implantation of lead electrodes during surgery (Wagner et al., 2007), thereby avoiding risk of infection and elicitation of an immune response. Second, tFUS can offer a superior spatial resolution on a millimeter scale and perform a deeper stimulation (Vignon et al., 2006; Mueller et al., 2016). However, because of the absorption and refraction of the skull and other biological tissues in the head, the focal spot of tFUS diverges and the sound pressure attenuates (Sugiyama, 2015; Khanna, 2016), which is a key issue influencing precise focusing.

Based on the simulation computation, the attenuation of tFUS has been previously investigated. The results show that the skull is the most influential factor in the attenuation of ultrasound pressure (Song et al., 2020). In addition, the error of peak intracranial pressure was noted to be greater than 2.6% when the thickness of the skull changed by 0.1 mm (Robertson et al., 2017). It is known that, for the management of different neurological diseases, tFUS needs to stimulate different brain locations, and ultrasound waves need to pass through variable thicknesses of skull at different points. Moreover, even if the location is same, different people have different skull thicknesses. However, the attenuation of tFUS based on the differences in skull thickness has not yet been estimated using a mathematical model, which is critical for accurately controlling the dose of tFUS.

In the present study, for precise focusing of tFUS, a mathematical model of ultrasound attenuation with skull thickness was constructed. Both numerical simulations and real skull phantom experiments were conducted to investigate the tFUS attenuation of different head tissues and to test the performance of the mathematical model. In addition, the potential applications of this model for different initial sound pressures and different skulls were also explored.

## Materials and Methods

In this study, two real human skulls (No. 14 and 49) were acquired, which were supported by *Tianjin Medical College.* The skulls were obtained via voluntary donations for the purpose of medical practice.

In addition, a perfectly matched layer was set on the edge of the model to absorb the output ultrasound waves and prevent their reflection back to the model to cause interference. It should be noted that the acoustic wave propagation was required to be linear, and the amplitude of the shear wave in the tissue domain was needed to be much smaller than the amplitude of the pressure wave. Therefore, the non-linear effects and shear waves were ignored.

### Mathematical Model

To achieve precise focusing of tFUS, a mathematical model of ultrasound attenuation was constructed considering the skull thickness, which can be described as:


(1) $$\tau_{z}=61.85e^{-0.2537z}\times 100\%$$

(2) $$A_{z}=P_{\mathrm{water}}\times \left(1-\tau_{z}\right)$$

where z is the thickness of the skull, A_z_ is the corresponding transcranial ultrasound attenuation, P_water_ is the peak sound pressure in a pure water medium, and τ_z_ is the percentage of the sound pressure in a pure water medium to the sound pressure passing through the skull with a thickness of z and is defined as:


(3) $$\tau_{z}=\frac{P_{z}}{P_{\mathrm{water}}}\times 100\%$$

where P_z_ is the peak sound pressure when the skull thickness is z.

**FIGURE 1:**
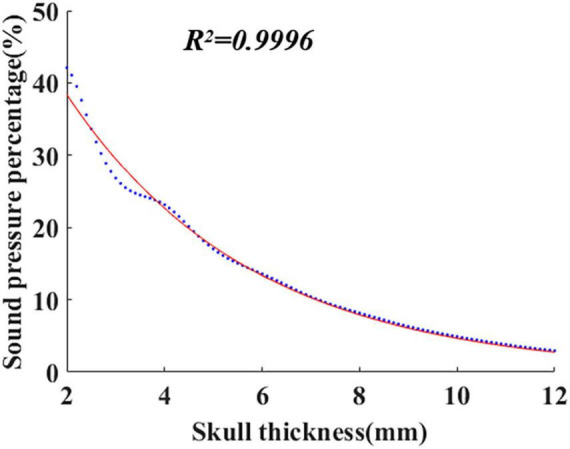
The peak sound pressure percentage fitting curve with skull thickness.

Equation (1) was established based on the system identification method. The finite element software COMSOL was used to simulate the ultrasonic propagation within the skull, and the corresponding sound pressure P_z_ was calculated with different skull thicknesses. Then, P_z_ was introduced into Equation (3) to calculate the sound pressure percentage τ_z_, and the obtained data are shown in Figure 1 (blue dots in the figure). As shown in Figure 1, the red curve is fitted by the least square method. The formula of the curve is the mathematical model of ultrasound attenuation with skull thickness, i.e., Equation (1). The coefficient of determination (*R*^2^) is 0.9996.

According to the proposed mathematical model, with knowledge of skull thickness, intracranial ultrasound attenuation can be directly calculated. In addition, the mathematical model is potentially suitable for different initial sound pressures using the sound pressure percentage instead of the sound pressure value.

In addition, based on the proposed mathematical model, the intracranial peak sound pressure can be further predicted as:


(4) $$P_{x2}=P_{x1}\times \frac{\tau_{x2}}{\tau_{x1}}$$

where x1 is the skull thickness of a certain site, P_x1_ is the corresponding intracranial peak sound pressure, τ_x1_ is the sound pressure percentage calculated by Equation (1), x2 is the skull thickness of another site, and P_x2_ is the intracranial peak sound pressure, which is to be predicted.

It can be seen that, with knowledge of intracranial sound pressure at a certain head site, the intracranial sound pressure at other sites can be predicted. Moreover, it should be noted that, as the prediction is a linear approximate calculation, the prediction accuracy depends on the thickness variation range. The smaller the thickness variation range, the higher the prediction accuracy.

### Simulation Experiment

**FIGURE 2:**
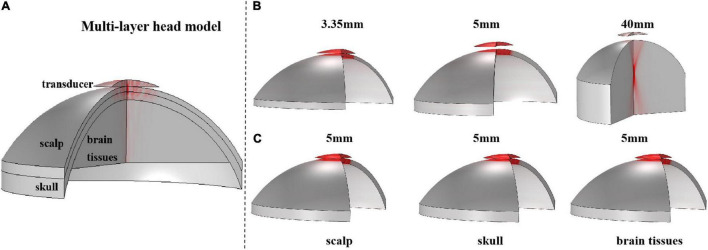
The 3-D structure of the multilayer head model and single-layer head tissue model. **(A)** Multilayer head model, including scalp, skull, and brain tissue, and the thickness is 3.35 and 5 mm and 4 cm, respectively. The top is a bowl-shaped ultrasound transducer. **(B)** 3-D structure of single-layer head tissue model with different thicknesses. The thickness of scalp, skull, and brain tissue is 3.35 and 5 mm and 4 cm, respectively. **(C)** 3-D structure of single-layer head tissue model with the same thickness. The thickness of scalp, skull, and brain tissue is unified to 5 mm.

First, based on the real head structure and biological tissue properties of the head region, a series of simulation models, including a 3-D multilayer head model and single-layer head tissue models were designed using finite element software COMSOL Multiphysics v5.4 (COMSOL, Burlington, MA). The 3-D multilayer head model is shown in Figure 2A. Consistent with the real skull phantom experiment, the multilayer head model included a 3.35-mm scalp and a 5-mm skull. The brain tissue was 4 cm thick. At the top of the model, a bowl-shaped ultrasound transducer was placed, which can simulate ultrasound waves with different sound pressures by adjusting the normal displacement of the transducer element. Two types of models were constructed for a single-layer head tissue model. As shown in Figure 2B, the single-layer head tissue model has different thicknesses. The thicknesses of the scalp, skull, and brain tissue were 3.35 and 5 mm and 4 cm, respectively. As shown in Figure 2C, another single-layer head tissue model was created with a uniform thickness. The thickness of the scalp, skull, and brain tissue was unified to 5 mm.

Thereafter, sound field calculation was implemented with the COMSOL software using the Pressure Acoustics, Frequency Domain (acpr) interface, which is a sound pressure finite element method solver using the homogeneous Helmholtz equation. An adaptive first-order triangular element mesh was used for the solver. The homogeneous Helmholtz equation is expressed as follows:


(5) $$\frac{\partial }{\partial r}\left[-\frac{r}{\rho_{c}}\left(\frac{\partial p}{\partial r}\right)\right]+r\frac{\partial }{\partial z}\left[-\frac{1}{\rho_{c}}\left(\frac{\partial p}{\partial z}\right)\right]-\left[{\left(\frac{\omega }{c_{c}}\right)}^{2}\right]\frac{\mathrm{rp}}{\rho_{c}}=0$$

where r and z are the radial and axial coordinates, p is the sound pressure, and ω is the angular frequency. Density ρ_c_ and sound velocity c_c_ have complex values and are used to represent the damping properties of the material. The material parameters are shown in Table 1. The main parameters were the speed of sound, density, and attenuation coefficient. From Table 1, it can be seen that the attenuation coefficient of the skull is 2,000 dB/m, and the sound speed is 4,080 m/s, which is much larger than that of the other tissues. For instance, the attenuation coefficient of the scalp is 68 dB/m, and the sound speed is 1,450 m/s. Similar to the scalp, the attenuation coefficient of brain tissue is 85 dB/m, and the sound speed is 1,552 m/s. For water, the default material properties were adopted in COMSOL. A focused ultrasound transducer of bowl shape was designed with a center frequency f0 of 1 MHz and diameter of 46 mm. The temperature T was set as 295.15 K.

**TABLE 1:** Head model material parameters.

Material	Speed of sound (m/s)	Attenuation coefficient (dB/m)	Density(g/cm^3^)
Scalp	1,450 (Greenleaf, 1986; Mingxi, 2010)	68 (Greenleaf, 1986; Mingxi, 2010)	955 (Jinhai, 2017)
Skull	4,080 (Greenleaf, 1986; Mingxi, 2010)	2,000 (Greenleaf, 1986; Mingxi, 2010)	1,658 (Jinhai, 2017)
Brain	1,552 (Samoudi et al., 2019)	85 (Jinhai, 2017)	1,046 (Samoudi et al., 2019)

### Phantom Experiment

**FIGURE 3:**
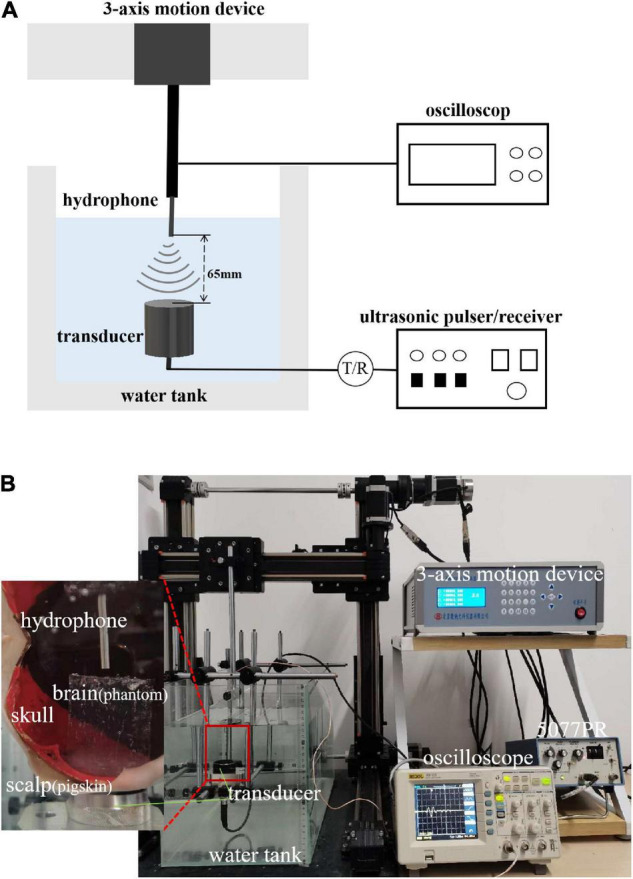
**(A)** Layout of experiment equipment. The experimental system consisted of a water tank, 3-axis motion device, hydrophone, ultrasound transducer, ultrasonic pulser/receiver, and oscilloscope. **(B)** Scene of experiment. The figure shows the equipment and its corresponding position. The relation of the equipment to the position of the samples (scalp, skull bones, and brain tissue) is also shown.

**FIGURE 4:**
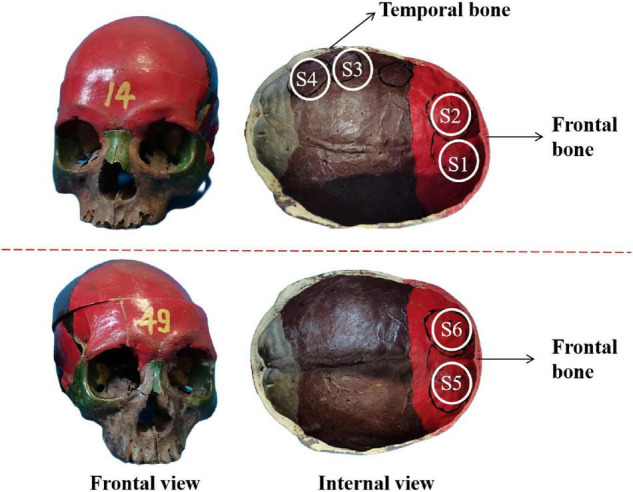
The frontal view and internal view of the real skull. S1–S4 and S5, S6 are selected as the experimental test points.

The experimental system is shown in Figures 3A,B. A single-element ultrasound transducer (Olympus, A392S, frequency: 1 ± 0.365 MHz, diameter: 46 mm PZT material) was used and immersed in a water tank. An ultrasonic pulser/receiver (Olympus, 5077PR, JP) was used to drive the ultrasound transducer. The ultrasound field was measured by a hydrophone mounted on a 3-D motion device. The signal measured by the hydrophone was displayed by an oscilloscope. The distance and time of ultrasound propagation was calculated due to the influence of reflected echo. As shown in Figure 4, two real human skulls (No. 14 and No. 49), supported by Tianjin Medical College, are used in this study. The skulls were used to evaluate the performance and explore the potential applications of the mathematical model. The following phantom experiments were conducted using the proposed system.

#### Ultrasound Measurement in the Water Tank

Most focused ultrasound applications in brain science are based on the ultrasonic characteristics measured in pure water. However, the ultrasonic characteristics in the brain are different from those in pure water. To quantitatively analyze the difference and prove the sound pressure attenuation of tFUS, a water tank experiment was conducted.

The sound pressure was chosen based on the literature on tFUS neuromodulation. For example, Yu et al. (2020) select a sound pressure of 0.8092 MPa in their experiment. Sound pressures of 0.5, 1, 2, and 4 MPa are used in another study (Gaur et al., 2020). Therefore, similar sound pressures were chosen for the present study. Corresponding to this sound pressure range, the single-element ultrasound transducer was driven by 5077PR with 100, 200, 300, and 400 V excitation voltage. The transducer was immersed in a water tank, and without the skull, the maximum pressure was measured using a hydrophone. The maximum pressure of the focal spot was 0.862, 1.931, 2.138, and 2.276 MPa, respectively.

#### Attenuation of Transcranial-Focused Ultrasound

In the phantom experiment, a real skull (No. 14), including the scalp, skull, and brain tissue, was used. The scalp was constructed with pigskin, and the brain tissue was constructed with a configured tissue phantom (made of acrylamide, methylene bisacrylamide, ammonium persulfate, egg white, and TEMED). The thicknesses of the scalp, skull, and brain tissue were 3.35 and 5 mm and 4 cm, respectively. The excitation voltage was 400 V, and without the skull, the corresponding maximum pressure of the focal spot was 2.276 MPa.

**FIGURE 5:**
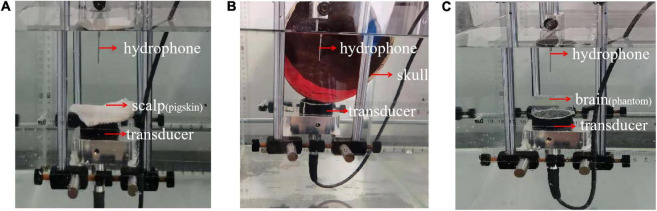
Phantom experimental photograph of different head tissues. **(A)** Pigskin, **(B)** skull, and **(C)** tissue phantom are placed on ultrasound transducer, respectively, and the peak sound pressure is measured by a hydrophone.

In addition, with a single-layer head tissue model, the effects of different head tissues were separately assessed in the phantom experiment. Consistent with the simulation experiment, the thicknesses of the scalp and skull were 3.35 and 5 mm, respectively. The thickness of the brain tissue was 4 cm. A photograph of the phantom experiment is shown in Figure 5. The ultrasound transducer was fixed in a tank filled with water with the pigskin (Figure 5A), skull (Figure 5B), and brain tissue phantom (Figure 5C) placed on it, and the sound pressure was measured using a hydrophone.

#### Verification of the Mathematical Model

Two real human skulls, No. 14 and No. 49, were used to verify the validity of the mathematical model. As shown in Figure 4, four test points are selected and called S1, S2, S3, and S4 in skull No. 14 with thicknesses of 3.5, 4.5, 5, and 6.5 mm, respectively. S1 and S2 were both located in the frontal bone, and the corresponding brain area is the frontal lobe. Two separate positions on the frontal bone were also selected in skull No. 49 to contrast with skull No. 14. The two positions were called S5 and S6, both having a thickness of 7.1 mm. The six sites of the skull (S1–S6) were measured under four different sound pressure conditions (0.862, 1.931, 2.138, and 2.276 MPa).

The positions of the ultrasound transducer, hydrophone, and real human skull are shown in Figure 5B. From bottom to top, the positions of the three are as follows: ultrasonic transducer, skull, and hydrophone. The different test points were placed above the transducer, and the peak sound pressure was measured with a hydrophone. The attenuation value was compared with the calculation of the mathematical model.

## Results

### Ultrasound Attenuation

#### Pure Water and Multilayer Head Model

**FIGURE 6:**
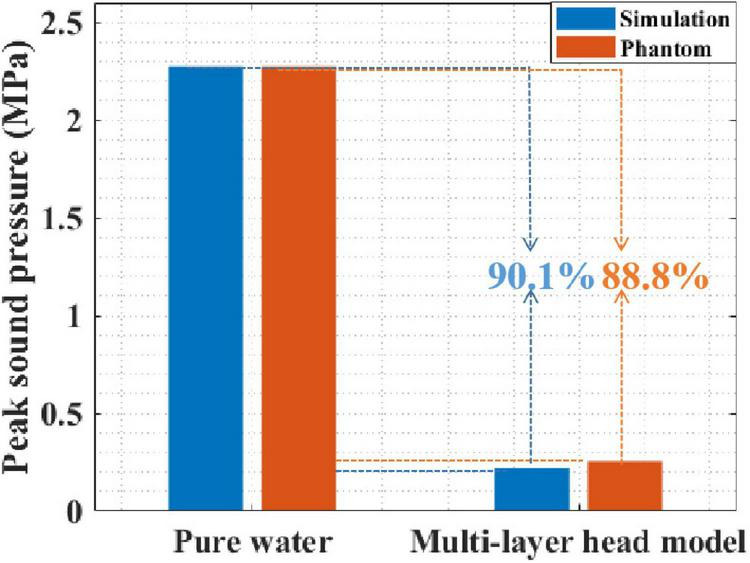
The sound pressure of both pure water and multilayer head model with simulation and phantom experiment.

First, the peak sound pressure attenuation of tFUS was investigated using simulation and phantom experiments. Figure 6 presents the peak sound pressure of both pure water and the multilayer head model. In pure water, the peak sound pressures in the simulation and phantom experiments were 2.276 and 2.276 MPa, respectively. In the multilayer head model, the peak sound pressures of the simulation and phantom experiments were 0.219 and 0.255 MPa, respectively. The simulation and phantom experiment results were consistent, which proved the effectiveness of the simulation and phantom experiments. Moreover, compared with that in pure water, the peak sound pressure in the multilayer head model was attenuated by 90.1% with simulation. The peak sound pressure was attenuated by 88.8% in the phantom experiment. Compared with that of pure water, the peak sound pressure of the multilayer head model was obviously reduced in both the simulation and phantom experiments.

**FIGURE 7:**
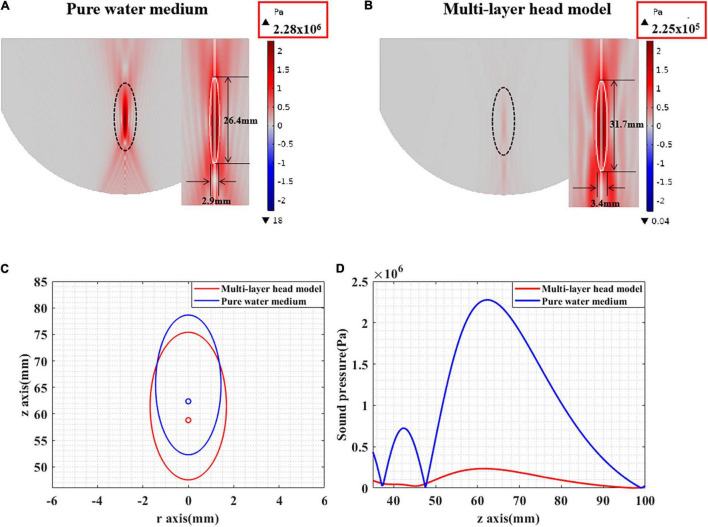
**(A)** The sound pressure distribution in pure water medium. **(B)** The sound pressure distribution of tFUS. **(C)** The FWHM distribution in pure water medium and tFUS. **(D)** The sound pressure value along the ultrasound propagation direction, including pure water medium and multilayer head model.

The ultrasound field distribution is presented in Figures 7A,B. It can be seen that, compared with that in pure water, the peak sound pressure is significantly attenuated in the multilayer head model. In addition, the focal spot [full width at half maximum (FWHM)] is diverged as shown in Figure 7C. The focal length is shortened, and the focal spot has moved forward as shown in Figure 7D. Experimental results show that the ultrasound field in the multilayer head model was significantly attenuated and cannot be effectively guided by the ultrasound field of pure water medium.

#### Different Head Tissues

**FIGURE 8:**
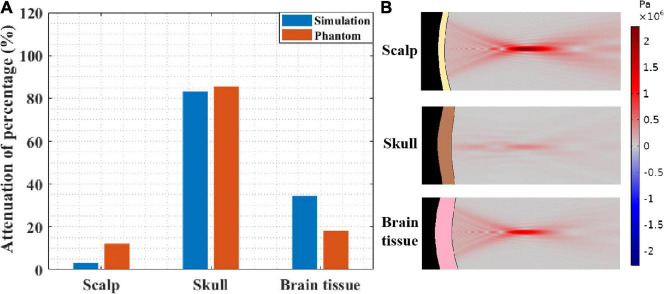
**(A)** Ultrasound attenuation percentage of different head tissues (thicknesses of scalp, skull and brain tissues are 3.35 and 5 mm and 4 cm, respectively). **(B)** The sound pressure distribution in scalp, skull, and brain tissue, respectively.

To further investigate the impact of different head tissues on ultrasound attenuation, experiments were conducted on the scalp, skull, and brain tissue. As shown in Figure 2B, the corresponding thicknesses are 3.35 and 5 mm and 4 cm, respectively, which are consistent with the head model parameters. Figure 8A shows the resulting ultrasound attenuation percentage. In the simulation experiment, the scalp caused an attenuation of 3.21% of the ultrasound peak sound pressure, the skull caused 83.22%, and the brain tissue caused 34.49% attenuation. In the phantom experiment, the scalp, skull, and brain tissue caused attenuation of 12.10, 85.45, and 18.20%, respectively. The head tissue ultrasound field distributions are shown in Figure 8B. Results show that the skull has the biggest influence on ultrasound propagation in the head, and the attenuation caused by it is 4.70 and 7.06 times that by the brain and scalp, respectively.

At the same time, it needs to be pointed out that, besides the difference in biological tissues of the head, the thickness of biological tissues is also different in different head tissue phantom experiments. This is also the reason why the influences of scalp and brain tissue are somewhat different despite both being soft tissues. Therefore, as shown in Figure 2C, the simulation experiment was implemented, in which the thickness of all the head tissues was unified to 5 mm. The corresponding ultrasound attenuation percentage results are displayed in Table 2. The results show that the skull caused an attenuation of 85.45% of the peak ultrasound sound pressure. The attenuation of scalp and brain tissues were very close, at 3.78 and 5.54%, respectively. These results further confirm that the skull is the main factor in tFUS attenuation, and therefore, it is critical to study the skull tissue for tFUS.

**TABLE 2:** With the same thickness, ultrasound attenuation percentage of different head tissues.

	Scalp	Skull	Brain tissue
Thickness (mm)	5	5	5
Peak focal pressure (MPa)	2.19	0.382	2.15
Attenuation (%)	3.78	85.45	5.54

### Mathematical Model Verification

**FIGURE 9:**
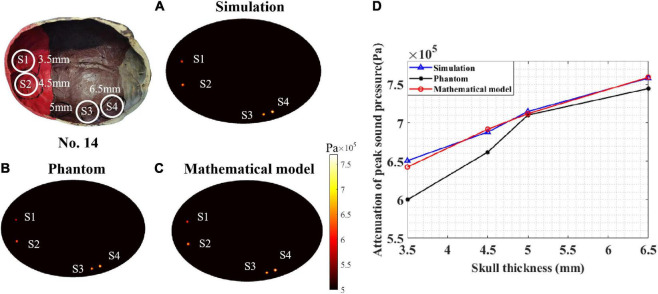
Peak sound pressure at different test points for skull No. 14. **(A)** Simulation experiment result. **(B)** Phantom experiment result and **(C)** mathematical model result. **(D)** The attenuation of peak sound pressure with different skull thicknesses. The thickness of test points S1, S2, S3, and S4 are 3.5, 4.5, 5, and 6.5 mm, respectively.

To test the performance of the proposed mathematical model, a sound pressure attenuation experiment of the real skull (No. 14) was conducted. As shown in Figure 9, four points (S1–S4) were tested with different thicknesses: 3.5, 4.5, 5, and 6.5 mm. According to the peak sound pressure measured in the water tank without the skull, the initial peak sound pressure was set to 0.862 MPa for both the phantom and COMSOL simulations. In the COMSOL simulation, the skull thickness coefficient was consistent with the test points.

The COMSOL simulation, phantom experiment, and mathematical model results are displayed in Figures 9A–C. It can be seen that, for different skull thicknesses, the attenuation has a significant difference. For the COMSOL simulation result (Figure 9A), test point S4 has a maximum attenuation value of 88%. The attenuation values of S3, S2, and S1 were 82.96, 79.85, and 75.52%, respectively. As the thickness of the skull increased, the attenuation of the peak sound pressure increased as shown in Figure 9D. This phenomenon was also observed in the phantom experiment (Figure 9B). The peak sound pressure attenuation was 86.40, 82.40, 76.80, and 69.60% in the four test points S4, S3, S2, and S1, respectively. Consistent with the results of the COMSOL simulation and phantom experiment, as the skull thickness increased, the attenuation increased for the mathematical model calculation. For the four test points, the corresponding sound pressure attenuations were 88.11, 82.60, 80.25, and 74.55%, respectively. Compared with the COMSOL simulation results, for the mathematical model, the maximum and minimum errors were 1.30 and 0.13%, respectively. Compared with the phantom experiment results, the corresponding maximum error and minimum error are 6.64 and 0.25%, respectively. Thus, it can be concluded that, compared with the simulation and phantom experiments, the mathematical model can effectively calculate the ultrasound attenuation for different skull thicknesses.

### Mathematical Model Applications

#### Application for Different Initial Sound Pressures

**FIGURE 10:**
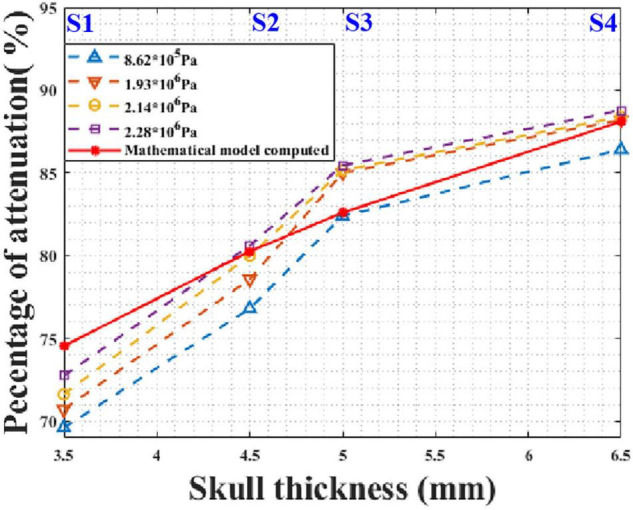
Peak sound pressure attenuation results for different initial sound pressures at different test points of skull No. 14.

In practical applications, tFUS needs to transmit ultrasound waves with different sound pressures. For instance, different sound pressures are required for different pathologies during ultrasound neuromodulation. It is critical that the model is applicable for different initial sound pressures. According to the phantom experimental conditions, the initial peak sound pressures of the focal spot were 0.862, 1.931, 2.138, and 2.276 MPa without the skull. The mathematical model and phantom experiment results are shown in Figure 10. It can be seen that, for different initial sound pressures, the phantom experiment results and model calculation results are in good agreement. As the initial sound pressure increased, the peak transcranial sound pressure increased. As the thickness of the skull increased, the attenuation of the peak sound pressure increased. Compared with the phantom experiment results, the maximum and minimum errors of the mathematical model were 6.64 and 0.11%, respectively. These results demonstrate that the mathematical model is applicable to transducers with different initial sound pressures.

#### Application for Different Skulls

**FIGURE 11:**
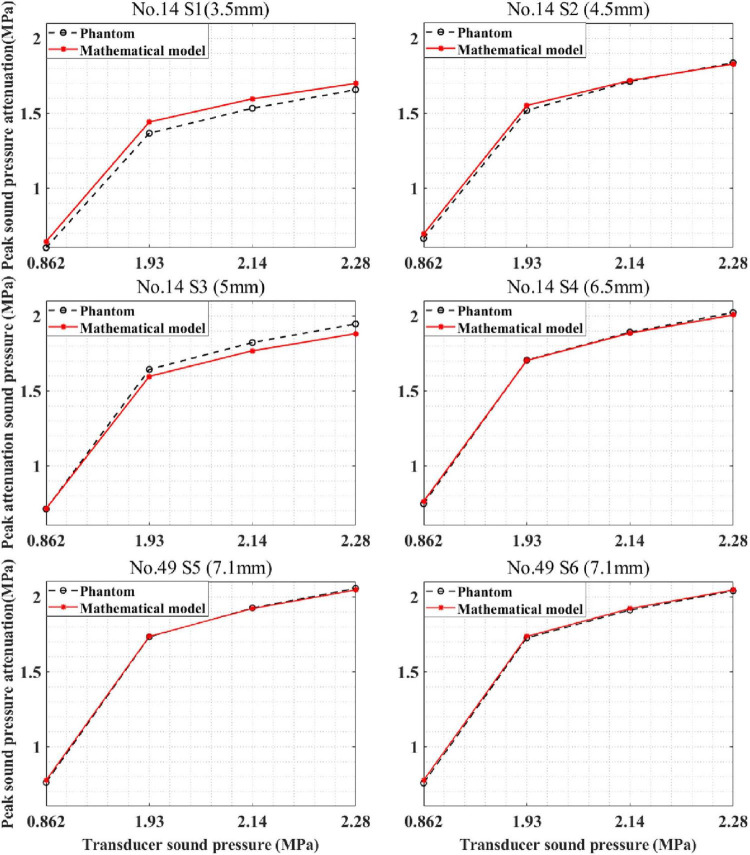
Peak sound pressure attenuation results of model prediction and phantom experiment for different skulls. S1–S4 are located at different positions of skull No. 14, and S5, S6 are located on skull No. 49.

The devised mathematical model was used to calculate the sound pressure attenuation of different skulls. The mathematical model and phantom experiment results are shown in Figure 11. For skull No. 14, the correlation coefficients between the phantom experiment and model calculation results were 99.95, 99.96, 99.99, and 99.99% for the four test points. Compared with the phantom experiment results, for the mathematical model, the maximum errors were 6.64, 4.30, and 0.25% 1.94%, respectively. For skull No. 49, the correlation coefficient between the phantom experiment and model calculation results was more than 99.99% for the two test points with the same thickness (7.1 mm). The difference in the correlation coefficient is due to the measurement error of the phantom experiment. The computational maximum errors are 1.99 and 2.44%. The results indicate that the mathematical model accurately calculates the sound pressure attenuation for different skulls and has the potential to be used by different people in practical applications.

#### Application for Multilayer Head Model

**FIGURE 12:**
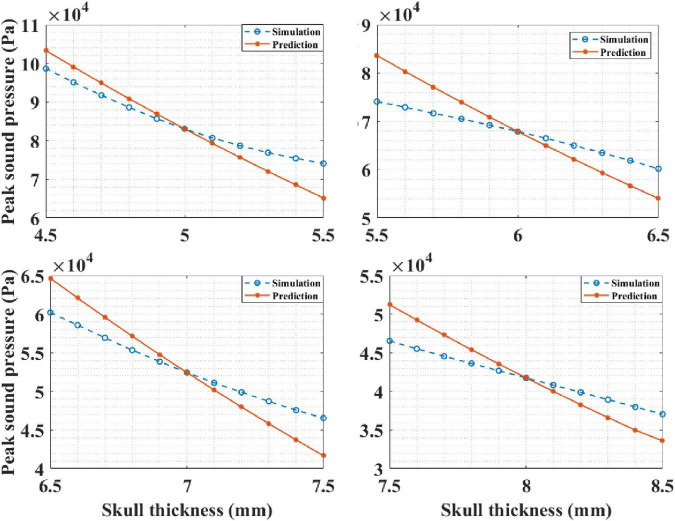
The intracranial sound pressure results of mathematical model prediction and phantom experiment for different known skull thicknesses.

Based on the knowledge of intracranial sound pressure at a certain head site, our mathematical model, Equation (4), was able predict the intracranial sound pressure at the other head sites. It should be noted that the skull thickness was the only variable here, and the thickness of other biological tissues remained unchanged. In the simulation experiment, the thickness of the skull was known, and it was set as 5, 6, 7, and 8 mm in each run. Then, the corresponding intracranial peak sound pressure of the other sites is predicted by the mathematical model. The results are presented in Figure 12. It can be observed that the predicted curve is consistent with the simulation results. The correlation coefficient between the prediction and simulation results was 99.34% when the known skull thickness was 5 mm. The corresponding correlation coefficients between the prediction and simulation results were 99.60, 99.91, and 99.93% for the other three known thicknesses. The results show that the mathematical model can accurately predict the intracranial sound pressure at other sites and can contribute to the further development and improvement of tFUS.

## Discussion

In this study, influence of the different tissues of the head on tFUS attenuation was investigated. Compared with that of the pure water medium, the peak sound pressure of tFUS was attenuated by 90.1% in the simulation and 88.8% in the phantom experiment. Phantom and simulation experiment results show that the skull can cause a much larger attenuation compared with other head tissues, the calculated attenuation being 85.45 and 83.22%, respectively. Thus, skull thickness can be regarded as the main factor for ultrasound attenuation in tFUS. These results demonstrate that the focused ultrasound field in pure water medium is not applicable for tFUS applications in clinical practice, and it is meaningful to explore the tFUS field characteristics.

Furthermore, to achieve tFUS precise focusing, a mathematical model of ultrasound attenuation is proposed. Both simulation and real skull experiments validate that the mathematical model can effectively calculate the ultrasound attenuation for different skull thicknesses (with a maximum error of 6.64%). In addition, the mathematical model is suitable for different initial pressures and skulls. This mathematical model can also predict the intracranial sound pressure at a certain head site, and the correlation coefficient was higher than 0.99. In summary, the presented experimental results validate that the mathematical model can effectively calculate tFUS attenuation, which is valuable for the precise application of tFUS as well as further research in this area.

At the same time, it should be noted that this study has a few limitations. Some characteristics have not been considered in the simulation, such as the viscoelasticity and non-uniformity of tissues, the non-linear effects, and shear waves of the ultrasound field. In the future, research focusing on the tissues and ultrasound field characteristics need to be considered to construct a precise model of tFUS.

## Data Availability Statement

The original contributions presented in the study are included in the article/supplementary material, further inquiries can be directed to the corresponding author/s.

## Ethics Statement

Ethical review and approval was not required for the study on human participants in accordance with the local legislation and institutional requirements. Written informed consent for participation was not required for this study in accordance with the national legislation and the institutional requirements.

## Author Contributions

XS and DM conceptualized the study and were responsible for project administration. JG conceived, designed, performed the experiments, and wrote the manuscript. JG and XC supervised the experiment. XS and JG analyzed the data and were responsible for data curation. XS reviewed and edited the manuscript. MX reviewed the manuscript. All authors contributed to the article and approved the submitted version.

## Conflict of Interest

The authors declare that the research was conducted in the absence of any commercial or financial relationships that could be construed as a potential conflict of interest.

## Publisher’s Note

All claims expressed in this article are solely those of the authors and do not necessarily represent those of their affiliated organizations, or those of the publisher, the editors and the reviewers. Any product that may be evaluated in this article, or claim that may be made by its manufacturer, is not guaranteed or endorsed by the publisher.

## References

[B1] Deffieux T. Younan Y. Wattiez N. Tanter M. Pouget P. Aubry J. F. (2013). Low-intensity focused ultrasound modulates monkey visuomotor. *Behav. Curr. Biol.* 23 2430–2433. 10.1016/j.cub.2013.10.029 24239121

[B2] Gaur P. Casey K. M. Kubanek J Li N Mohammadjavadi M Saenz Y Glover G. H Bouley D. M Pauly K. B. (2020). Histologic safety of transcranial focused ultrasound neuromodulation and magnetic resonance acoustic radiation force imaging in rhesus macaques and sheep. *Brain Stimul.* 13 804–814. 10.1016/j.brs.2020.02.017 32289711 PMC7196031

[B3] Greenleaf J. A. (1986). *Tissue Characterization with Ultrasound.* Boca Raton: CRC Press.

[B4] Jana B. Biswas R. Nath P. K. (2020). Smartphone-Based Point-of-Care System Using Continuous-Wave Portable Doppler. *IEEE Trans. Instrum. Meas.* 69 8352–8361. 10.1109/TIM.2020.2987654

[B5] Jinhai N. (2017). *Ultrasound Principles and Biomedical Engineering Applications.* Shanghai: Shanghai Jiao Tong University Press.

[B6] Khanna V. K. (2016). *Deep brain stimulation in Implantable Medical Electronics*, Switzerland: Springer International Publishing, 309–329.

[B7] Kranjec J. Beguš S. Drnovšek J. Geršak G. (2014). Novel Methods for Noncontact Heart Rate Measurement: a Feasibility Study. *IEEE Trans. Instrum. Meas.* 63 838–847. 10.1109/TIM.2013.2287118

[B8] Lee W. Kim H. C. Jung Y. Chung Y. A. Song I. U. Lee J. H. (2016). Transcranial focused ultrasound stimulation of human primary visual cortex. *Sci. Rep.* 6:34026. 10.1038/srep34026 27658372 PMC5034307

[B9] Legon W. Ai L. Bansal P. Mueller J. K. (2018). Neuromodulation with single-element transcranial focused ultrasound in human thalamus. *Hum. Brain Mapp.* 39 1995–2006. 10.1002/hbm.23981 29380485 PMC6866487

[B10] Mingxi W. (2010). *Biomedical Supersonics.* Beijing: Science press.

[B11] Mueller J. Legon W. Opitz A. Sato T. F. Tyler W. J. (2014). Transcranial focused ultrasound modulates intrinsic and evoked EEG dynamics. *Brain Stimul.* 7 900–908. 10.1016/j.brs.2014.08.008 25265863

[B12] Mueller J. K. Ai L. Bansal P. Legon W. (2016). Computational exploration of wave propagation and heating from transcranial focused ultrasound for neuromodulation. *J. Neural. Eng.* 13:056002. 10.1088/1741-2560/13/5/056002 27464603

[B13] Mueller J. K. Ai L. Bansal P. Legon W. (2017). Numerical evaluation of the skull for human neuromodulation with transcranial focused ultrasound. *J. Neural. Eng.* 14:066012. 10.1088/1741-2552/aa843e 28777075

[B14] Oluigbo C. O. Salma A. Rezai A. R. (2012). Deep brain stimulation for neurological disorders. *IEEE Rev. Biomed. Eng.* 5 88–99. 10.1109/RBME.2012.2197745 23231991

[B15] O’Shea J. Walsh V. (2007). Transcranial magnetic stimulation. *Curr. Biol.* 17 R196–R199. 10.1016/j.cub.2007.01.030 17371754

[B16] Robertson J. Martin E. Cox B. Treeby B. E. (2017). Sensitivity of simulated transcranial ultrasound fields to acoustic medium property maps. *Phys. Med. Biol.* 62 2559–2580. 10.1088/1361-6560/aa5e98 28165334

[B17] Samoudi M. A. Van Renterghem T. Botteldooren D. (2019). Computational modeling of a single-element transcranial focused ultrasound transducer for subthalamic nucleus stimulation. *J. Neural. Eng.* 16:026015. 10.1088/1741-2552/aafa38 30572313

[B18] Song X. Chen X. Guo J. Xu M. Ming D. (2021). Living rat SSVEP mapping with acoustoelectric brain imaging. *IEEE Trans. Biomed. Eng.* 69 75–82. 10.1109/TBME.2021.3087177 34101579

[B19] Song X. Guo J. Chen X. Ke Y. Ming D. (2020). *Investigation of Transcranial Focused Ultrasound Attenuation with Multilayer Head Model.* Italy: IEEE Int. Ultrasonics Symp, 1–4. 10.1109/IUS46767.2020.9251452

[B20] Sugiyama K. (2015). Deep Brain Stimulation for Neurological Disorders. *IEEE Rev. Biomed. Eng.* 5 88–99. 10.1109/RBME.2012.2197745 23231991

[B21] Tufail Y. Matyushov A. Baldwin N. Tauchmann M. L. Georges J. Yoshihiro A. (2010). Transcranial pulsed ultrasound stimulates intact brain circuits. *Neuron* 66 681–694. 10.1016/j.neuron.2010.05.008 20547127

[B22] Vignon F. Aubry J. F. Tanter M. Margourn A. Fink M. (2006). Adaptive focusing for transcranial ultrasound imaging using dual arrays. *J. Acoust. Soc. Am.* 120 2737–2745. 10.1121/1.2354073 17139734

[B23] Wagner T. Valero-Cabre A. Pascual-Leone A. (2007). Noninvasive human brain stimulation. *Annu. Rev. Biomed. Eng.* 9 527–565. 10.1146/annurev.bioeng.9.061206.133100 17444810

[B24] Yu K. Liu C. Niu X. He B. (2020). Transcranial Focused Ultrasound Neuromodulation of Voluntary Movement related Cortical Activity in Humans. *IEEE Trans. Biomed. Eng.* 68 1923–1931. 10.1109/TBME.2020.3030892 33055021 PMC8046844

[B25] Zhou Y. Song X. Wang Z. He F. Ming D. (2021). Multisource Acoustoelectric Imaging with Different Current Source Features. *IEEE Trans. Instrum.* 70 1–1. 10.1109/TIM.2020.3021496

